# Retrospective Single‐Centre Evaluation of Alternative D‐Dimer Thresholds in the Evaluation of Suspected Acute Pulmonary Embolism

**DOI:** 10.1155/emmi/3892586

**Published:** 2026-09-01

**Authors:** Anum Saad, Hannah Raye, David Manners

**Affiliations:** ^1^ St John of God Midland Public Hospital, Midland, Perth, Western Australia, Australia; ^2^ Curtin Medical School, Curtin University, Bentley, Perth, Western Australia, Australia, curtin.edu.au

## Abstract

This retrospective evaluation examined the theoretical impact of alternative D‐dimer thresholds (age‐adjusted and 1000 µg/L) in patients aged ≥ 50 years who underwent computed tomography pulmonary angiography (CTPA) after D‐dimer testing. Among 291 patients with positive D‐dimer and subsequent CTPA, the age‐adjusted threshold maintained 100% observed sensitivity (95% CI 89.7%–100%), while the 1000‐µg/L threshold missed three pulmonary emboli (sensitivity 91.2%, 95% CI 76.3%–98.1%). Applying these thresholds would have reduced CTPA use by 21.7% and 46.7%, respectively. These findings suggest that age‐adjusted D‐dimer thresholds may improve diagnostic efficiency in selected older patients; however, the absence of prospectively recorded pretest probability, verification bias and a lack of follow‐up prevents conclusions about clinical safety.

## 1. Introduction

Acute pulmonary embolism (PE) is a potentially life‐threatening condition with a broad clinical presentation and substantial diagnostic complexity. D‐dimer, a fibrin degradation product, is widely used as an initial test to exclude PE in patients with low clinical pretest probability [1]. The risk of 3‐month thromboembolic events following a negative D‐dimer in low‐risk patients is less than one percent [2].

D‐dimer is a continuous biomarker, and several diagnostic strategies incorporate clinical pretest probability to optimise threshold selection. These include the Wells score, revised Geneva score, ADJUST PE (age‐adjusted thresholds), the YEARS algorithm and the PEGeD strategy [3–5]. These strategies aim to reduce posttest probability to below approximately 1.8%, a threshold generally considered acceptable for ruling out PE [1].

Although the standard threshold for a positive D‐dimer is 500 µg/L, recent studies suggest that age‐adjusted thresholds or a 1000‐µg/L threshold may reduce imaging while maintaining acceptable failure rates [4, 5]. International guidelines now acknowledge the role of age‐adjusted thresholds, but this has not been widely adopted in Australian practice [1, 6].

This study evaluates the theoretical impact of applying age‐adjusted and 1000‐µg/L thresholds in patients aged ≥ 50 years who underwent computed tomography pulmonary angiography (CTPA) after D‐dimer testing. Given the retrospective design and absence of pretest probability assessment, the study aims to describe potential imaging reduction rather than establish diagnostic safety.

## 2. Methods

### 2.1. Study Design, Setting and Participants

A retrospective cohort study was conducted at St John of God Midland Public Hospital (SJGMPH). Ethics approval was obtained using awaiver of consent on 21 May 2021 (SJOG Healthcare HREC Ref 1750). The radiology database was used to identify consecutive patients that underwent CTPA scans requested by the SJGMPH Emergency Department. Patients were included if they had a D‐dimer performed within 24 h prior to CTPA. Patients aged < 50 years were excluded because age‐adjusted thresholds apply only to older adults. The study period predates the COVID‐19 pandemic and therefore does not reflect COVID‐associated VTE patterns. Pretest probability was not prospectively documented, and D‐dimer ordering cannot reliably be assumed to indicate low pretest probability. This limitation is acknowledged and limits safety conclusions.

### 2.2. D‐Dimer Assay, Thresholds, Reference Standard and Missing Data

D‐dimer testing was performed using a fibrinogen equivalent unit (FEU) assay and reported in µg/L. Three prespecified thresholds were evaluated: 500 µg/L (standard), age‐adjusted (age in years × 10 µg/L) and 1000 µg/L, based on alternative thresholds suggested by prior prospective studies [3–5]. CTPA reports were used as the reference standard, with subsegmental PE considered positive. The reporting radiologist was not blinded to the D‐dimer results. Indeterminate scans underwent blinded review by an experienced chest radiologist. Patients without a D‐dimer within 24 h of CTPA were excluded, and no missing data imputation was performed.

### 2.3. Statistical Analysis

Sensitivity, specificity, PPV and NPV (the latter two presented in supporting information) were calculated using the Wilson method for confidence intervals. Due to verification bias and missing pretest probability, these metrics reflect performance within this selected cohort only. Full 2 × 2 contingency tables for each threshold are provided in Supporting Tables s1–s3.

## 3. Results

Of 801 CTPAs reviewed, 291 patients aged ≥ 50 years had a D‐dimer performed within 24 h and were included. Thirty‐four patients (11.6%) had PE. A participant flow diagram is provided in the online supplement.

Using the age‐adjusted threshold, sensitivity remained 100% (95% CI 89.7%–100%), with specificity increasing to 24.5%. Applying this threshold would have reduced CTPA use by 22% (63/291). No PE cases fell below the age‐adjusted threshold, although the confidence interval remains wide due to the small number of events.

Using the 1000‐µg/L threshold, sensitivity decreased to 91.2% (95% CI 76.3%–98.1%), with specificity increasing to 51.8%. At this threshold, 46.7% (136/291) of CTPAs would have been avoided. Three PE cases fell below the 1000‐µg/L threshold, all of whom had moderate or high clinical risk factors, including recent PE, recent hospitalisation with immobility or multiple sclerosis with prior PEs. These false negatives are clinically important and highlight the limitations of applying this threshold without clinical probability assessment.

Full contingency tables for each threshold are presented in Supporting Tables s1–s3.

## 4. Discussion

This retrospective evaluation suggests that applying age‐adjusted or 1000‐µg/L D‐dimer thresholds could theoretically reduce CTPA use in older patients. These findings align with international evidence suggesting that modified thresholds may improve diagnostic efficiency. However, several major methodological limitations prevent conclusions about diagnostic safety and require careful interpretation.

A key limitation is verification bias. Only patients with positive D‐dimer who underwent CTPA were included, while patients with negative D‐dimer who were not scanned were excluded. This prevents calculation of true sensitivity and specificity for diagnostic accuracy and may inflate sensitivity estimates. Additionally, this cohort only includes patients already chosen for imaging; therefore, it does not represent a true diagnostic population. Equally important is the absence of prospectively recorded pretest probability. D‐dimer use in suspected PE is validated for use only in patients with low or intermediate clinical probability, and without rigorous pretest scoring, the study cannot determine whether initial D‐dimer was used appropriately.

The three false‐negative cases at the 1000‐µg/L threshold are clinically significant. Although this threshold substantially increases imaging efficiency, it does so at the cost of reduced diagnostic safety. These cases underscore the importance of integrating clinical probability with D‐dimer interpretation and highlight why higher thresholds cannot be applied in isolation.

The age‐adjusted threshold demonstrated 100% observed sensitivity in this cohort, but the confidence interval remains wide due to the small number of PE cases. This finding should therefore be interpreted as promising but underpowered rather than confirmatory.

The absence of follow‐up means that delayed or missed VTE events cannot be assessed, precluding definitive conclusions about clinical safety. Potential system‐level benefits, such as reduced imaging burden and improved patient flow, require prospective evaluation and should not be inferred from this retrospective dataset. While reduced imaging may decrease detection of clinically insignificant subsegmental PE or other incidental findings, this study was not designed to evaluate overdiagnosis nor potential reductions in contrast exposure and radiation.

Comparison with prior studies must also be cautious. ADJUST PE, YEARS and PEGeD were prospective diagnostic management studies with structured pretest probability assessment and mandated follow‐up. In contrast, the current study is a retrospective service evaluation describing theoretical imaging reduction, yet the results are similar to a previous Australian retrospective study [7]. Table 1 summarises these studies, but direct comparison is limited by differences in design, population and methodology.

**TABLE 1:** Overview of current and previous studies investigating D‐dimer thresholds.

Study	Setting	Design	Population	PE prevalence (%)	Key findings
Current study (SJGMPH)	Single Australian hospital	Retrospective cohort	≥ 50 years, suspected PE	11.60	Age‐adjusted: 22% fewer CTPAs, 0% observed FN; 1000 µg/L: 46.7% fewer CTPAs, 3 FN (91.2% sensitivity)
ADJUST‐PE [3]	19 European hospitals	Prospective	≥ 18 years	19	Age‐adjusted threshold increased proportion ruled out without imaging, especially ≥ 75 years
YEARS [4]	12 Dutch hospitals	Prospective	≥ 18 years	13	YEARS algorithm reduced imaging by 14% with low failure rate
PEGeD [5]	Canadian centres	Prospective	≥ 18 years	7.40	Low‐risk patients with D‐dimer < 1000 µg/L had very low failure rate
Woo & Thien [7]	Single Australian hospital	Retrospective	≥ 18 years	12.70	Age‐adjusted threshold reduced imaging by 27.9%

*Note:* SJGMPH: St John of God Midland Public Hospital, PEGeD: pulmonary embolism graduated D‐dimer study, ADJUST PE: age‐adjusted D‐dimer cut‐off levels to rule out pulmonary embolism.

Abbreviations: CTPA, computed tomography pulmonary angiogram; FN, false negatives; PE, pulmonary embolism.

Overall, this study provides local observational data suggesting that age‐adjusted D‐dimer thresholds may reduce imaging in selected older patients. However, due to verification bias, missing pretest probability, a lack of follow‐up and clinically important false negatives, these findings should be interpreted as exploratory or hypothesis‐generating. Future research should include prospective Australian cohorts with structured pretest probability assessment, follow‐up for missed events and evaluation of patient‐centred outcomes. Shared decision‐making frameworks may also help clinicians balance efficiency and acceptable failure rates.

## 5. Conclusion

Age‐adjusted D‐dimer threshold in suspected PE may improve theoretical diagnostic efficiency in older patients; however, verification bias, missing pretest probability, a lack of follow‐up and clinically important false negatives prevent conclusions about safety. Prospective studies with structured clinical probability assessment are required.

## Author Contributions

David Manners conceived the study and performed the analysis; Anum Saad and Hannah Raye undertook data collection; David Manners and Anum Saad drafted the manuscript.

## Funding

Open‐access publishing was facilitated by Curtin University, as part of the Wiley–Curtin University agreement via the Council of Australasian University Librarians.

## Conflicts of Interest

The authors declare no conflicts of interest.

## Supporting Information

Additional supporting information can be found online in the Supporting Information section.

## Supporting information


**Supporting Information** Participant flow diagram and full 2 × 2 contingency tables (Tables S1–S3) showing D‐dimer threshold performance within the selected cohort. These tables provide sensitivity, specificity, PPV and NPV calculations for the standard, age‐adjusted and 1000‐µg/L thresholds.

## Data Availability

The data used to support the findings of this study are available from the corresponding author upon request.
