# Supplementary material for: Retrospective Single‐Centre Evaluation of Alternative D‐Dimer Thresholds in the Evaluation of Suspected Acute Pulmonary Embolism
Source: Emerg Med Int. 2026 Sep 1;2026:3892586. doi: 10.1155/emmi/3892586 (PMC13535148; doi:10.1155/emmi/3892586)
Supplement: Supplementary file 1 [file EMMI-2026-3892586-s001.docx]

**Supplementary File: STARD Participant Flow Diagram and Diagnostic Performance Tables**

**STARD Participant Flow Diagram:**

CTPA: Computed Tomography Pulmonary Angiogram. PE: Pulmonary Embolism

**Diagnostic performance of D‑dimer thresholds in patients aged ≥50 years undergoing CTPA:**

This supplement provides full 2×2 contingency tables for each D‑dimer threshold evaluated in the study cohort. These tables allow transparent calculation of sensitivity, specificity, positive predictive value (PPV), and negative predictive value (NPV) within this selected population of patients who had both a positive D‑dimer and a CTPA performed.

Because only D‑dimer–positive patients who underwent CTPA were included, these tables reflect performance **within this selected cohort only** and do not represent true diagnostic accuracy in an unselected population.

**Table S1. Standard D‑dimer threshold (500 µg/L)**

*All included patients had D‑dimer ≥500 µg/L.*

| **D‑dimer ≥500 µg/L** | **PE+** | **PE–** | **Total** |
| --- | --- | --- | --- |
| **Positive** | 34 | 257 | 291 |
| **Negative** | 0 | 0 | 0 |
| **Total** | 34 | 257 | 291 |

**Interpretation:**

- Sensitivity: 100% (assumed within this cohort)
- Specificity: 0%
- PPV: 11.6%
- NPV: Not applicable (no negative tests)

**Table S2. Age‑adjusted D‑dimer threshold (age × 10 µg/L)**

| **Age‑adjusted threshold** | **PE+** | **PE–** | **Total** |
| --- | --- | --- | --- |
| **Positive** | 34 | 194 | 228 |
| **Negative** | 0 | 63 | 63 |
| **Total** | 34 | 257 | 291 |

**Interpretation:**

- Sensitivity: 100%
- Specificity: 24.5%
- PPV: 14.9%
- NPV: 100%
- CTPA potentially avoided: 22% (63/291)

**Table S3. Fixed D‑dimer threshold (1000 µg/L)**

| **D‑dimer ≥1000 µg/L** | **PE+** | **PE–** | **Total** |
| --- | --- | --- | --- |
| **Positive** | 31 | 124 | 155 |
| **Negative** | 3 | 133 | 136 |
| **Total** | 34 | 257 | 291 |

**Interpretation:**

- Sensitivity: 91.2%
- Specificity: 51.8%
- PPV: 20.0%
- NPV: 97.6%
- CTPA potentially avoided: 46.7% (136/291)
- False negatives: 3 clinically significant PE cases
